# Supplementary material for: Causal Relationship between Aging and Anorexia Nervosa: A White-Matter-Microstructure-Mediated Mendelian Randomization Analysis
Source: Biomedicines. 2024 Aug 16;12(8):1874. doi: 10.3390/biomedicines12081874 (PMC11351342; doi:10.3390/biomedicines12081874)
Supplement: Supplementary file 1 [file biomedicines-12-01874-s001.zip › Supplementary Figure/Figure S7/Single SNP plot of MR analysis for ubm-b-1964 to AN.pdf]

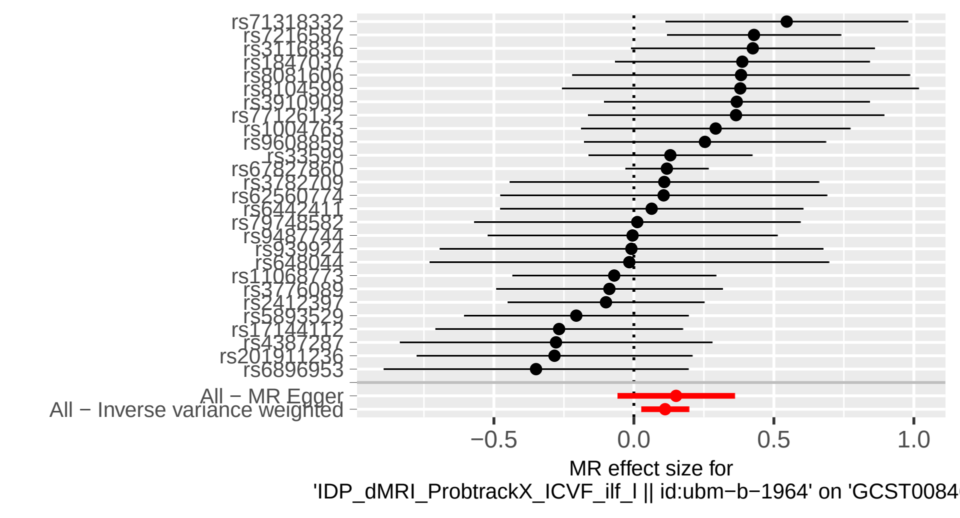
rs71318332
rs7216587
rs3116836
rs1847037
rs8081606
rs8104599
rs3910909
rs77126132
rs1004763
rs9608859
rs33599
rs67827860
rs3782709
rs62560774
rs6442411
rs79748582
rs9487744
rs939924
rs648044
rs11068773
rs3776089
rs2412397
rs5893529
rs17144112
rs4387287
rs201911236
rs6896953
All − MR Egger
All − Inverse variance weighted
−0.5
0.0
0.5
1.0
MR effect size for
'IDP_dMRI_ProbtrackX_ICVF_ilf_l || id:ubm−b−1964' on 'GCST0084
